# Treatment of a Cold

**Published:** 1892-03

**Authors:** 


### TREATMENT OF A COLD.

A brisk walk is recommended for breaking up a cold. The person threatened should put on extra clothing, and walk hard and fast until he is in a free perspiration. Then, while still heated up, he should go home, quickly undress and get into a warm bed, and take a glass full of hot water or hot lemonade. Where this course is pursued, the chances are many that all the threatening signs will have disappeared the following morning.

A "sweat" always acts admirably in any kind of cold, and if not brought about by exercise, it ought to be produced by some of the many other means known to housekeepers. If the cold is in the head, the following treatment promises well: Add a teaspoonful of powdered camphor to a pitcher of boiling water. Over this place a cone made of thick paper or pasteboard, and hold the nose and mouth over the small opening. The vapor arising from the water is charged with camphor, and will speedily relieve many of the disturbing symptoms. It should be inhaled for four or five minutes at a sitting, and three such treatments are usually sufficient to arrest the most rebellious "cold in the head."

But many of the victims of this trouble must be out and about. A snuff is more convenient for them. A very good one can be made up as follows: Menthol, three grains; powdered boracic acid, one drachm; subnitrate of bismuth and powder benzoin, each 1½ drachms. A good sized pinch may be snuffed up five or six times a day.

